# Pseudo Atrium? A Rare Presentation of Interventricular Septal Dissection Extending Into the Left Atrium

**DOI:** 10.1016/j.jaccas.2023.101940

**Published:** 2023-08-02

**Authors:** Yuta Kato, Mitsuyoshi Hadase

**Affiliations:** Department of Cardiology, Saiseikai Shiga Hospital, Shiga, Japan

**Keywords:** echocardiography, interventricular septal dissection, ventricular septal perforation

## Abstract

Interventricular septal dissection is a rare complication of myocardial infarction. In cases with few left-to-right shunts in the ventricular septal perforation, interventricular septal dissection expands in the chronic phase. It is rare for the interventricular septal dissection to extend from the ventricular septum to the left atrial free wall. (**Level of Difficulty: Intermediate.**)

An 86-year-old woman presented to the emergency department with general fatigue and chest pain for several days. She had a holosystolic murmur, Levine III/VI, best heard at the left lower sternal border. The electrocardiogram showed negative T waves in leads II, III, and aVF. Her serum high-sensitivity troponin I level was 2,606 pg/mL (reference value, <16.6 pg/mL), and the creatine kinase-myocardial band level was 59 U/L (reference value, <25 U/L). A transthoracic echocardiogram revealed the compression of the left atrium by a pseudoatrium, and we detected interventricular septal dissection (IVSD) (Figures 1A and 1B, Videos 1 and 2). The basal inferoseptal was an aneurysm and other wall motions were normal. Blood flow to the pseudoatrium was confirmed; however, the inflow area could not be identified (Video 2). Contrast-enhanced computed tomography scan revealed a wide ventricular septal defect at the basal inferoseptal left ventricular wall, and an IVSD was shown to exist between the left and right ventricles. The IVSD was contiguous with the pseudoaneurysm and partially thrombosed (Figures 1C and 1D). The pseudoatrium compressing the left atrium was found to be a continuous pseudoaneurysm from the IVSD. Coronary angiography revealed total occlusion of the right coronary artery with the collateral vessels from the left coronary artery. Transthoracic echocardiogram and contrast-enhanced computed tomography scan did not show traffic from the IVSD to the right ventricle, so we performed left ventriculography. Left ventriculography revealed inflow into the pseudoaneurysm owing to IVSD and into the right ventricle owing to septal perforation (Video 3). Based on these findings, we concluded that myocardial infarction caused IVSD, which formed a pseudoaneurysm in the left atrial free wall, and that ventricular septal perforation (VSP) resulted from the IVSD. The next day, the patient underwent emergency surgery, and the interventricular septal defect was closed with bovine pericardial double patching. In this case, the patient’s IVSD and pseudoaneurysm were thrombosed and regressed (Figures 1E and 1F). One month later, this patient was discharged after rehabilitation.

**Figure 1:**
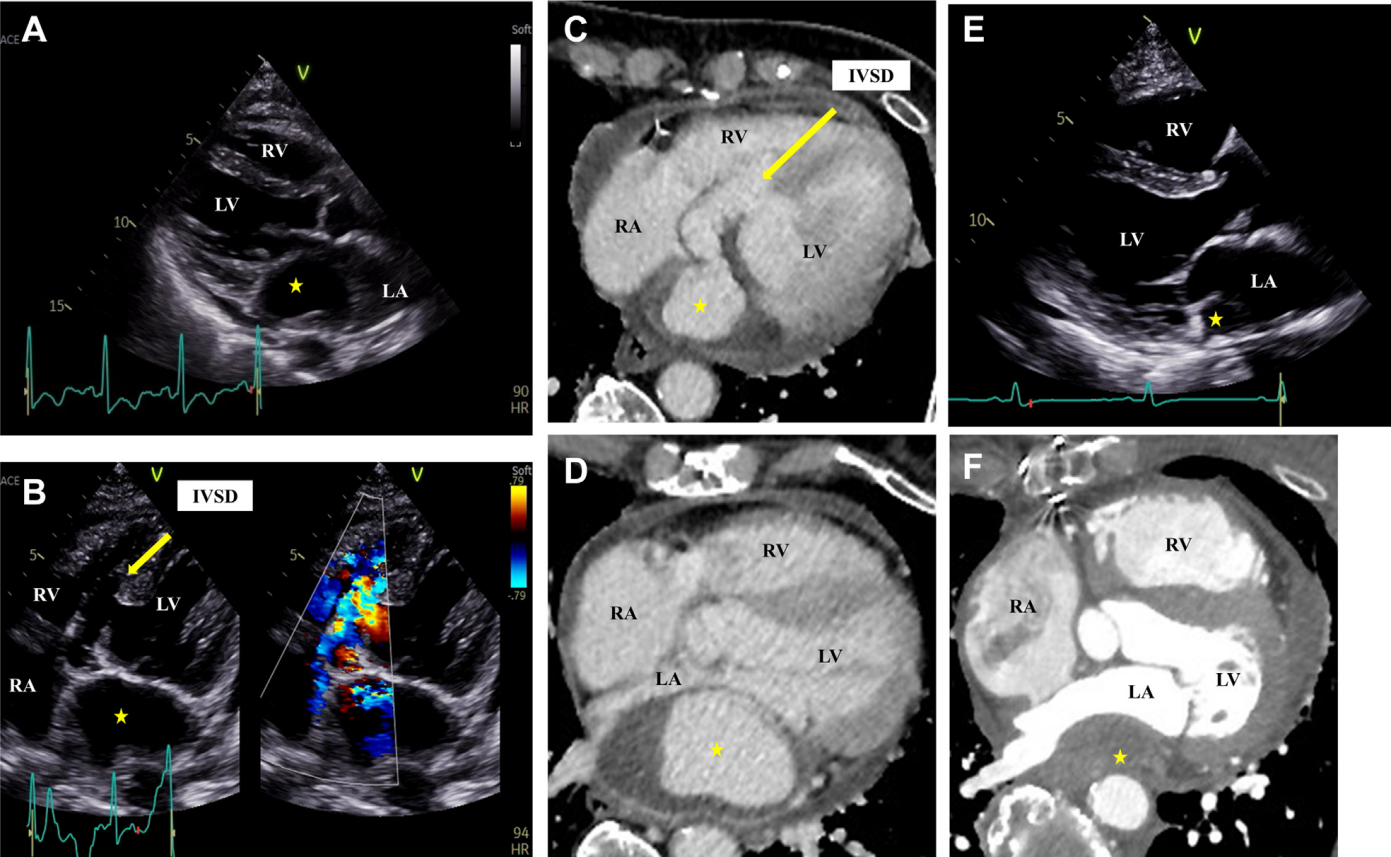
Imaging of the Interventricular Septal Dissection and Pseudoaneurysm Before and After Operation **(A, E)** Transthoracic echocardiogram, left sternal border approach and long-axis view. **(A)** The pseudoaneurysm **(yellow star)** compressed the left atrium (Video 1). **(B)** Transthoracic echocardiogram, apical 4-chamber view highlighting the IVSD as well as the RA, RV, LA, and LV (Video 2). **(C, D, F)** Contrast-enhanced computed tomography axial view. **(C)** IVSD extending to the left atrium. **(D)** The left atrial outflow tract is significantly compressed by the pseudoaneurysm. **(E, F)** Four months after surgery, the pseudoaneurysm was regressed **(yellow star)**. IVSD = interventricular septal dissection; LA = left atrium; LV = left ventricle; RA = right atrium; RV = right ventricle.

## Discussion

VSP occurs in approximately 0.3% of patients after acute myocardial infarction. This disease requires prompt diagnosis and urgent surgical intervention because it causes hemodynamic failure and is fatal within a short period of time. Surgical closure is the most common treatment, with a reported mortality rate of >90% with medical therapy. The 30-day mortality rate for surgical closure was 61%.^1^ Currently, the use of the Impella (Abiomed) is reported to be effective in delaying the time of surgery. Because the boundary between the infarcted area and the healthy area is unclear in the acute phase, it is believed that the fibrosis around the perforation site will increase over time, which is effective in preventing residual short circuit and improving the prognosis.^2^ IVSD is a rare complication of myocardial infarction that is associated mostly with the VSP of the posterior type. IVSD forms a pseudoaneurysm, and it is suspected that VSP develops when a left-to-right shunt occurs. In cases with few left-to-right shunts in the VSP, IVSD expands in the chronic phase.^3^

In this case, we consider that the hemodynamic compromise did not occur because of the small volume of the left-to-right shunt. There have been no reports of IVSD extending to the left atrium. In this case, the left atrial outflow tract was severely compressed (Figure 1D). This would have been extremely dangerous because, if the pseudoaneurysm were to enlarge, it could have resulted in obstructive shock of the left atrium. We experienced a rare presentation of IVSD extending into the left atrium.

## Funding Support and Author Disclosures

The authors have reported that they have no relationships relevant to the contents of this paper to disclose.

## Appendix

Video 1 Transthoracic Echocardiogram Left sternal border approach and long-axis view with the left atrium compressed by the pseudoaneurysm.

Video 2 Transthoracic Echocardiogram Apical 4-chamber view.

Video 3 Left Ventriculography (30° Right Anterior Oblique) The contrast in the left ventricle flows from the interventricular septal dissection into the pseudoaneurysm and right ventricle.
